# Diagnosis of invasive candidiasis by enzyme-linked immunosorbent assay using the N-terminal fragment of *Candida albicans *hyphal wall protein 1

**DOI:** 10.1186/1471-2180-7-35

**Published:** 2007-04-21

**Authors:** Ana Laín, Natalia Elguezabal, Sonia Brena, Juan Carlos García-Ruiz, Amalia del Palacio, María D Moragues, José Pontón

**Affiliations:** 1 Departamento de Inmunología, Microbiología y Parasitología, Facultad de Medicina y Odontología, Universidad del País Vasco, Apartado 699, E-48080 Bilbao, Vizcaya, Spain; 2 Servicio de Hematología, Hospital de Cruces, Baracaldo, Vizcaya, Spain; 3 Unidad de Micología, Departamento de Microbiología, Hospital Universitario 12 de Octubre, Madrid, Spain; 4 Departamento de Enfermería I, Universidad del País Vasco. Barrio Sarriena s/n, 48940 Lejona, Vizcaya, Spain

## Abstract

**Background:**

The diagnosis of invasive candidiasis is difficult because there are no specific clinical manifestations of the disease and colonization and infection are difficult to distinguish. In the last decade, much effort has been made to develop reliable tests for rapid diagnosis of invasive candidiasis, but none of them have found widespread clinical use.

**Results:**

Antibodies against a recombinant N-terminal fragment of the *Candida albicans *germ tube-specific antigen hyphal wall protein 1 (Hwp1) generated in *Escherichia coli *were detected by both immunoblotting and ELISA tests in a group of 36 hematological or Intensive Care Unit patients with invasive candidiasis and in a group of 45 control patients at high risk for the mycosis who did not have clinical or microbiological data to document invasive candidiasis. Results were compared with an immunofluorescence test to detect antibodies to *C. albicans *germ tubes (CAGT). The sensitivity, specificity, positive and negative predictive values of a diagnostic test based on the detection of antibodies against the N-terminal fragment of Hwp1 by immunoblotting were 27.8 %, 95.6 %, 83.3 % and 62.3 %, respectively. Detection of antibodies to the N-terminal fragment of Hwp1 by ELISA increased the sensitivity (88.9 %) and the negative predictive value (90.2 %) but slightly decreased the specificity (82.6 %) and positive predictive values (80 %). The kinetics of antibody response to the N-terminal fragment of Hwp1 by ELISA was very similar to that observed by detecting antibodies to CAGT.

**Conclusion:**

An ELISA test to detect antibodies against a recombinant N-terminal fragment of the *C. albicans *germ tube cell wall antigen Hwp1 allows the diagnosis of invasive candidiasis with similar results to those obtained by detecting antibodies to CAGT but without the need of treating the sera to adsorb the antibodies against the cell wall surface of the blastospore.

## Background

Invasive *Candida *infections are a serious and often fatal nosocomial disease in immunocompromised patients. The diagnosis of invasive candidiasis is difficult because there are no specific clinical manifestations of the disease and colonization and infection are difficult to distinguish. Conventional microbiological methods, which include observation of the infecting fungus by histopathology and culture, usually lack both sensitivity and specificity and sometimes they require invasive procedures that can not be accomplished because of the clinical conditions of the patients [1]. As a result of these problems, therapy is often initiated late in the course of infection, resulting in substantial morbidity and mortality [2].

During the last two decades, much effort has been made to develop reliable tests for rapid diagnosis of invasive candidiasis leading to appropriate therapy. These techniques include the detection of fungal nucleic acid by PCR [3], (1→3) β-D-glucan [4], D-arabinitol [5] and a number of circulating antigens and antibodies [6]. However, all techniques have limitations and, at the moment, none of them have found widespread clinical use.

*Candida albicans *is a fungus that can grow in either the yeast form or the hyphal form, and the ability to germinate and form hyphae may be a factor for the virulence of this organism *in vivo *[7,8]. Our group has previously reported that the detection of antibodies specifically directed to antigens expressed on the *C. albicans *germ tube surface (CAGT) by indirect immunofluorescence has shown a sensitivity of 79–89 % and a specificity of 91–100% for the diagnosis of invasive candidiasis on both competent and immunocompromised patients [6,9,10]. A number of antigens specifically expressed on the *C. albicans *germ tube cell wall have been identified recently including hyphal wall protein 1 (Hwp1) [11], Als3p [12], Ece1p [13] and Hyr1p [14]. As it has been studied in other mycoses [15], these antigens can be obtained in recombinant form, allowing the detection of antibodies against them.

In this study, we report the development of both an immunoblotting and an enzyme-linked immunosorbent assay (ELISA) for the serodiagnosis of invasive *Candida *spp. infections by detecting specific antibodies against a recombinant N-terminal fragment of Hwp1. Results were compared with an immunofluorescence test to detect antibodies to CAGT.

## Results

### Characterization of the recombinant N-Hwp1 fragment

**Figure 1:**
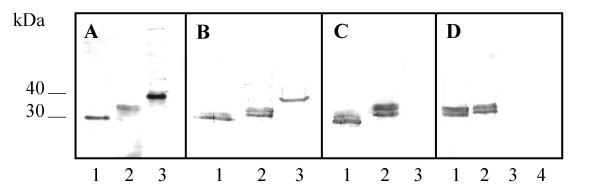
Western blots of 10% slab gels loaded with Hwp1 antigens of *C. albicans *stained with silver stain (panel A), an anti-Myc monoclonal antibody (panel B, lane 1), an anti-HSV monoclonal antibody (panel B, lanes 2 and 3), a polyclonal antiserum against the purified recombinant N-Hwp1 fragment (panel C) and human sera (panel D). Gels in panels A-C were loaded with rHwp1N13KV-c~myc (lane 1), N-Hwp1 (lane 2) and int-Hwp (lane 3). Panel D shows the reactivity of N-Hwp1 with sera from two patients with invasive candidiasis (lanes 1 and 2) and from two patients without invasive candidiasis (lanes 3 and 4). Molecular masses (in kDa) of standard proteins are listed to the left of the gels.

The purified N-Hwp1 fragment obtained in this study yielded a protein with an apparent molecular mass of 38 kDa, while the purified int-Hwp1 yielded a protein of 42 kDa (Fig. 1A). Both recombinant fragments reacted by immunoblotting with the anti HSV·tag^® ^monoclonal antibody (Fig. 1B). However, the anti HSV·tag^® ^monoclonal antibody revealed that the purified N-Hwp1 migrated as a doublet of 36–38 kDa. The purified rHwp1N13KV-c~myc fragment yielded a protein of 34 kDa (Fig. 1A). A monoclonal antibody against the c-Myc·tag revealed that the purified rHwp1N13KV-c~myc fragment migrated also as a doublet of 34–36 kDa (Fig. 1B). Differences in molecular mass between N-Hwp1 and rHwp1N13KV-c~myc proteins are explained by the different size of the Hwp1 fragments and the different tags used.

A rabbit anti-N-Hwp1 antiserum reacted with both N-terminal Hwp1 fragments (Fig. 1C). The antiserum revealed the existence of two closely located bands in both N-Hwp1 and rHwp1N13KV-c~myc fragments with molecular masses of 36–38 kDa and 34–36 kDa, respectively (Fig. 1B). The specificity of the anti-N-Hwp1 antiserum was confirmed by the lack of reactivity with the int-Hwp1 fragment (Fig. 1C).

### Detection of antibodies by immunoblotting

The reactivity of the N-Hwp1 was tested by immunoblotting with 342 sera from 81 different patients. The N-terminal fragment was recognized as a double band of 36–38 kDa by sera from10 patients from group 1 (27.8 %) (Fig. 1D, Table 1). In four patients, the antibody response against N-Hwp1 was detected after the blood culture positive for *Candida*. Group 1 patients with antibodies against the recombinant N-Hwp1 fragment were infected with *C. albicans *and *C. glabrata *(Table 1). Sera from patients infected with *C. tropicalis, C. krusei, C. guilliermondii, C. parapsilosis *and *C. utilis *did not recognize the recombinant N-Hwp1 fragment by this technique. The majority of group 2 patients did not react by Western Blotting (Fig. 1D). However, two patients from group 2 (4.4 %) had antibodies that reacted with the recombinant N-Hwp1 fragment (Table 2). None of the sera recognized the int-Hwp1 fragment.

The sensitivity, specificity, positive and negative predictive values of a diagnostic test based on the detection of antibodies against the N-Hwp1 fragment by immunoblotting were 27.8 %, 95.6%, 83.3 % and 62.3 %, respectively.

### Detection of antibodies by indirect immunofluorescence

Antibodies to CAGT were detected in 35 patients with invasive candidiasis (group 1) (97.2 %). These patients had titers of antibodies to CAGT ranging 1:20 to 1:20480, and in 29 patients (80.6 %), the titer was ≥ 1:160. Group 1 patients showing antibodies to CAGT ≤ 1:160 were mainly infected by non-*C. albicans *species, including *C. parapsilosis*, *C. guilliermondii, C. krusei *and *C. tropicalis *(Table 1). Only 4 out of 45 (8.9 %) patients without evidence of invasive candidiasis (group 2) had antibodies to CAGT at titer ≥ 1:160 (Table 2).

Considering a cutoff value of = 1:160, the sensitivity, specificity, positive and negative predictive values of a diagnostic test based on the detection of antibodies to CAGT were 80.6 %, 91.1 %, 87.9 % and 85.4 %, respectively.

### Detection of antibodies by ELISA

In an attempt to increase the low sensitivity of the detection of antibodies against the N-Hwp1 fragment by immunoblotting, we developed and ELISA assay for the diagnosis invasive candidiasis. The cutoff value was defined as the mean plus 3 times the standard deviation of the relative absorbance values of the sera obtained from patients without evidence of candidiasis. Relative absorbance values above the cutoff were considered positive whereas results under the cutoff were negative. Thirty-two out of 36 patients from group 1 (88.9 %) were positive by the ELISA test (Table 1). Two patients infected with *C. albicans *and one patient each infected with *C. guilliermondii *and *C. krusei *were negative by this assay (Table 1). Eight out of 45 patients from group 2 tested (17.8 %) showed false positive results by ELISA (Table 2). Group I patients showed higher amounts of antibodies against N-Hwp1 fragment than patients from group II, since the mean plus standard deviation were 1.7 ± 0.7 and 0.7 ± 0.4, respectively (P < 0.01).

The sensitivity, specificity, positive and negative predictive values of a diagnostic test based on the detection of antibodies to the N-Hwp1 fragment by ELISA were 88.9 %, 82.6 %, 80.0 % and 90.2 %, respectively. Patients infected with *C. albicans *were more likely to have antibodies to the N-Hwp1 fragment above the cutoff value. In fact, if only patients infected with *C. albicans *were taken into consideration, the sensitivity and negative predictive value of the test increased to 91.3 % and 94.9 %, while the specificity remained unaltered.

**Figure 2:**
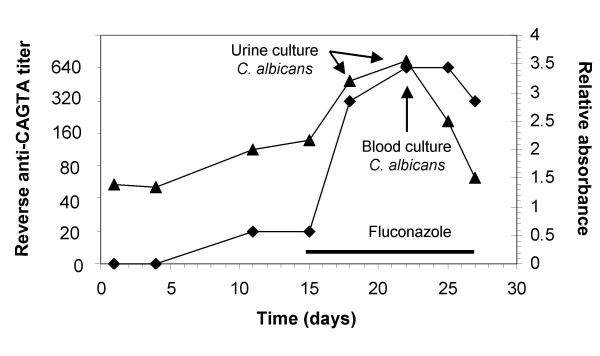
Kinetics of antibodies to Hwp1 (▲) and to CAGT (◆) of patient 17. Antibody levels ≥ 160 were considered positive for CAGT and ≥ 1.08 for Hwp1. Arrows point to the days where cultures of urine and blood yielded *C. albicans*. The bar shows the days the patient was treated with fluconazole.

In a subgroup of 14 group 1 patients, the availability of serial serum samples allowed to study the kinetics of antibody response to the N-Hwp1 fragment by ELISA and to CAGT. Antibody titers became positive before the diagnosis of invasive candidiasis was made in 9 patients by ELISA and in 6 patients by indirect immunofluorescence. Interestingly, the kinetics of antibody response to the N-Hwp1 fragment of by ELISA was very similar to that observed by indirect immunofluorescence and antibody titers showed a constant rise around the time the clinical and microbiological evidence of invasive candidiasis existed and then fell below the cutoff value if the patient responded to antifungal therapy. The kinetics of antibody response in a patient with invasive candidiasis is shown in Fig. 2.

**Figure 3:**
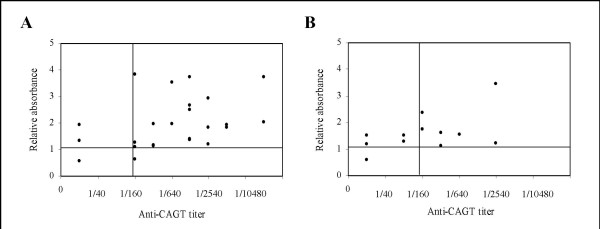
Scatterplot showing the relationship between the results obtained by ELISA and indirect immunofluorescence in patients infected with *C. albicans *(A) and in patients infected with non-*albicans Candida *species (B). The proposed cut-off values for indirect immunofluorescence and ELISA are indicated by the horizontal and vertical lines, respectively.

The relationship between the results obtained by ELISA and indirect immunofluorescence is shown in Figure 3. In patients infected with *C. albicans*, results obtained by both techniques showed a good agreement since only three patients (12.5 %) were positive by only one technique. In patients infected with non-*albicans Candida *species, the agreement was lower since four patients (33.3 %) were positive by ELISA but negative by indirect immunofluorescence. Antibody titers obtained with both techniques were lower for patients infected with non-*albicans *species than for those infected with *C. albicans*.

### Homology between *C. albicans *Hwp1 and other *Candida *proteins

**Figure 4:**
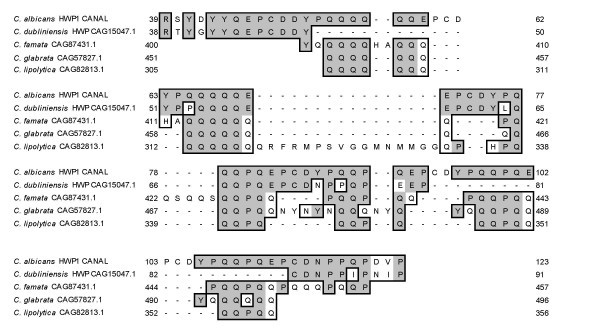
Alignment of sequences of proteins of five species of *Candida*. Regions of homology with HWP1 of *Candida albicans *are shown within boxes. Shadowed residues stand for sequence identity. Numbers to the left and right indicate the location within the amino acid sequence.

The presence in patients infected by non-*albicans Candida *species of antibodies that cross-react with *C. albicans *N-Hwp1 may be explained by the existence of common epitopes in *C. albicans *Hwp1 and other proteins present in non-*albicans Candida *species. To test this hypothesis we searched the GenBank database for homology between *C. albicans *Hwp1 and other *Candida *proteins. Interestingly, the region encoding the first 85 amino acid residues of Hwp1 showed homology with the sequence of proteins of *C. dubliniensis, C. famata, C. glabrata *and *C. lipolytica *(Fig. 4).

## Discussion

Due to the commensal nature of members of genus *Candida*, the key factor in the laboratory diagnosis of invasive candidiasis is the differentiation between infection and colonization. From a serological point of view, this differentiation may be facilitated if different types of antibodies are detected in each situation. Our group has previously reported the detection of antibodies to CAGT, which are usually detected in patients with invasive candidiasis at titers ≥ 160 and are absent or detected at very low titers in patients without invasive candidiasis despite of being colonized by *Candida *spp. [6,10,18]. The basis for this discrimination lies on the different antigenic composition of blastospores and germ tubes of *C. albicans*, since germ tubes but not blastospores express a high molecular weight antigen that induces an antibody response [20]. Due to the mannoproteic nature of the antigen, the detection of antibodies to CAGT requires the adsorption of antibodies directed against mannan since they are ubiquitous in human sera [21].

Identification of genes encoding antigens specific for the hyphal phase of *C. albicans *has opened new ways to study the usefulness of these antigens in the serological diagnosis of invasive candidiasis. For example, they can be obtained in recombinant form which, in addition to the availability of highly standardized reagents, will be non-glycosylated when they have been expressed in *E. coli*. The lack of glycosylation of these recombinant antigens is important because the adsorption of the sera to remove the anti-mannan antibodies will not be necessary, an important improvement when the ELISA test is compared to the detection of antibodies to CAGT.

*C. albicans HWP1 *is one of the phase-specific genes most studied. The glycoprotein encoded by this gene is specifically expressed on the cell wall surface of germ tubes and hyphae of *C. albicans *[22], and may be required for virulence in systemic candidiasis [23]. Its location on the cell wall surface may facilitate the production of antibodies against this protein by the *C. albicans*-infected host. Since the protein has a surface-exposed N-terminal domain that binds antibodies while the carboxyl terminus is most probably covalently integrated in cell wall [11], we selected that fragment of Hwp1 for our serological studies. The molecular mass of the N-Hwp1 was determined to be 38 kDa, much larger than the calculated 22 kDa. The same discrepancy was observed with the rHwp1N13KV-c~myc fragment used as control, since it yielded a protein of 34 kDa when the predicted molecular mass was 20.5 kDA. The lack of agreement between the predicted molecular mass and the SDS-PAGE-determined molecular mass has been reported previously and is related to the high proline content of Hwp1 [11]. The peculiar migration of the N-terminal fragments of Hwp1 in SDS-PAGE gels seems to be also responsible for the doublet revealed in the Western blots by the anti-N-Hwp1 antiserum and by sera from patients with invasive candidiasis. Both bands of each doublet are likely to be the same protein since they also reacted with the monoclonal antisera against the tag sequences of N-Hwp1 and rHwp1N13KV-c~myc proteins. Interestingly, a proline-rich antigen from *Coccidioides immitis *expressed in *E. coli *also migrated as a doublet in SDS-PAGE gels [24].

The diagnostic potential of the detection of antibodies against the N-Hwp1 fragment was studied by two different techniques: immunoblotting and ELISA. While detection of antibodies by immunoblotting showed a very poor sensitivity and negative predictive values, detection of antibodies against the N-terminal fragment of Hwp1 by ELISA notably increased the diagnostic validity, with sensitivity, specificity, positive and negative predictive values of 88.9 %, 82.6 %, 80.0 % and 90.5 %, respectively. Interestingly, these results were very close to those obtained by detecting antibodies to CAGT by indirect immunofluorescence. The kinetics of antibody response to CAGT antigens was also similar to that of antibodies against the recombinant protein. In some patients, antibody titers showed a constant rise in association with the clinical and microbiological diagnosis of invasive candidiasis and then decreased and eventually fell under the cut-off level if the patient responded to antifungal therapy. These results are in agreement with previously reported results detecting antibodies to CAGT by indirect immunofluorescence [6,10,18,25-27]. The relationship between the results obtained by detecting antibodies to N-Hwp1 and CAGT was higher in patients infected with *C. albicans *than in patients infected with non-*albicans Candida *species. In both cases, the majority of the discrepancies were due to the highest sensitivity of the ELISA, since six patients were positive by ELISA and negative by indirect immunofluorescence. Four of the six patients were infected with non-*albicans Candida *species, confirming previous observations that non-*albicans Candida *species induce lower titers of antibodies to CAGT [26-28].

False negative results by indirect immunofluorescence were observed in a group of patients who were infected by non-*albicans Candida *species. These results are in agreement with previously published results [26,27]. This is despite evidence that most clinically relevant non-*albicans Candida *species including *C. stellatoidea*, *C. guilliermondii*, *C. tropicalis*, *C. parapsilosis*, and *C. krusei *grown at 37°C have antigens that cross-react with those present in the *C. albicans *germ tube cell wall [28]. Interestingly, the ELISA test demonstrated that antibodies to the N-terminal fragment of *C. albicans *Hwp1 are detected in patients with infections by non-*albicans Candida *species including *C. parapsilosis*, *C. tropicalis*, *C. utilis*, *C. glabrata *and *C. dubliniensis*. The reasons for this reactivity are presently unknown since Hwp1 is exclusively synthesized during hyphal growth of *C. albicans *[11]. However, most clinically relevant *Candida *species, including *C. glabrata*, can produce pseudomycelium [29,30] and it has been recently reported that *HWP1 *mRNA may also arise from pseudohyphal growth forms in *C. albicans*, or possibly from yeast forms, although experimental data to support the presence of *HWP1 *mRNA in yeast forms has not been reported [31]. It may be possible that under certain conditions, some non-*albicans Candida *species express antigens that elicit antibodies that cross-react with *C. albicans *Hwp1. In agreement with this, Moran et al. [32] have recently identified in *C. dubliniensis *an ORF of 1266 bp with homology to *C. albicans *Hwp1, especially in the region encoding the first 50 residues of each protein, and we have observed that the region encoding the first 85 amino acid residues of Hwp1 shows homology with the sequence of proteins of a number of non-*albicans Candida *species including *C. dubliniensis, C. famata, C. glabrata *and *C. lipolytica*.

The existence of antibodies that cross-react with *C. albicans *Hwp1 in patients infected by non-*albicans Candida *species does not seem to decrease the specificity of the ELISA test developed in this study, since these antibodies seem to be present mostly in patients with invasive candidiasis. On the contrary, the existence of this cross-reactivity can be used to increase the sensitivity of the test, especially in a time when infections by non-*albicans Candida *species are increasing [33]. However, further studies investigating the induction of *C. albicans *Hwp1-like antigens by non-*albicans Candida *species are warranted.

The high sensitivity and specificity showed by the ELISA test developed in this study to detect antibodies against the N-terminal fragment of Hwp1 in the diagnosis of invasive candidiasis seem to be useful to detect the low levels of antibodies that may be present in hematological patients. However, an increase in both parameters might be obtained by detecting antibodies against other *C. albicans *germ tube specific antigens such as Als3p [12], Ece1p [13] and/or Hyr1p [14]. A mixture of all these antigens may be used in the same ELISA in order to optimize the result. Alternatively, an increase in the sensitivity can be obtained by combining the detection of antibodies to the N-terminal fragment of Hwp1 with a test that detects *Candida *antigens.

## Conclusion

This study shows that is possible to develop an ELISA test that allows the diagnosis of invasive candidiasis by detecting antibodies to the N-terminal fragment of *C. albicans *Hwp1. This test is simpler to perform than the detection of antibodies to CAGT since it does not need the adsorption of the patient's serum and allows both an objective measurement and the automatization of the test.

## Methods

### Strains, plasmids and culture media

*Candida albicans *SC5314 [16] was used as fungal DNA source in this study and was grown in medium 199 (Sigma Chemical Co., St. Louis, Mo, USA) at 24°C and 120 rpm. *C. albicans *NCPF 3153 (National Collection of Pathogenic Fungi, Bristol, UK) was used in some experiments in order to remove antibodies against the cell wall surface of the blastospore.

*Escherichia coli *(DE3) pLacI (Novagen, Darmstadt, Germany) was used as the host strain for the expression of the recombinant plasmid (pTriEx-1^® ^vector, Novagen). *E. coli *cells were grown in Luria-Bertani (LB) medium (1% w/v Tryptone, 1% NaCl, 0.5 % yeast extract, pH 7.0) or LB agar plates (LB and 1.5% agar, pH 7.0) supplemented, when necessary, with carbenicillin (50 μg/ml) and chloramphenicol (34 μg/ml) (Sigma).

### Cloning, expression and purification of the N-terminal and internal fragments of recombinant Hwp1

Primers (5'-GATATCCTCTTATGATTACTATCAAGAACC-3' and 5'-GGAATTCCCGTTTGGAGTAGCTGGAGT-3') were designed in order to clone the N-terminal fragment of the *HWP1 *coding sequence (N-Hwp1, from nucleotide 118 to 600, without the signal sequence). The amplified PCR product was digested with *Eco*RI and *Eco*RV restriction enzymes (Promega, Madison, WI, USA) and purified using QIAquick PCR purification Kit (Qiagen, Valencia, CA, USA). The *HWP1 *fragment was cloned into the sites of pTriEx-1^® ^vector containing His·tag^® ^and HSV·tag^® ^sequences (Novagen) to enable the construction of a C-terminal tagged fusion protein. To confirm the sequence of the insert and the frame of the recombinant tagged protein, the plasmid was sequenced by using primers flanking the polylinker region of the pTriEx-1^® ^plasmid. An internal fragment of *HWP1 *(int-Hwp1, from nucleotide 592 to 1050) was cloned using primers 5'-GATATCCACTCCAAACATTCCTGCTACA-3' and 5'-GGAATTCCCAGTCAATGGACAGTAAGTGGT-3', as stated for N-Hwp1.

*E. coli *cells were transformed with the plasmids containing the fragments of the *HWP1 *gene and were grown overnight in LB medium containing both carbenicillin and chloramphenicol in order to make the medium selective for the recombinant strains, at 37°C and 180 rpm. The cultures were used as inoculum of fresh LB medium and incubated up to an OD_600 _of 0.7–0.8. At this moment, the expression of the Hwp1 recombinant fragments was induced with 0.5 mM isopropyl β-D-thiogalactoside (Sigma). After 3.5 h of induction, cells were harvested by centrifugation at 13,000 rpm for 1.5 min and resuspended in 0.01 M phosphate buffered saline pH 7.2 (PBS). Protein extracts were checked for the presence of the recombinant Hwp1 fragments by sodium dodecyl sulfate-polyacrilamide gel electrophoresis (SDS-PAGE).

The recombinant His_6_-tagged Hwp1 fragments were purified by nickelchelate affinity chromatography in accordance to manufacturer's instructions (Novagen). Fractions containing the purified polypeptides were pooled, dialyzed against PBS and stored at -20°C.

A purified recombinant Hwp1 N-terminal fragment (rHwp1N13KV-c~myc) provided by Dr. Paula Sundstrom (Dartmouth Medical School, Department of Microbiology and Immunology, Hanover, NH, USA) was used to confirm the identity of the recombinant N-Hwp1 fragment produced for this study. The rHwp1N13KV-c~myc fragment was cloned from nucleotide 121 to 576 and produced in *Pichia pastoris *using the pPICZ alphaC vector containing c~Myc and 6x-His tags at the carboxy terminus (Invitrogen, Carlsbad, CA) (unpublished results, personal communication).

### Patients

We retrospectively studied 342 sera, stored at -20°C until use, from 81 different adult hematological cancer or Intensive Care Unit patients at increased risk for invasive candidiasis. Patients were divided into two groups, according to clinical and microbiological diagnostic data. Group 1 included 36 patients (193 sera) with invasive candidiasis, which was proven by a positive *Candida *spp. blood culture (Table 1). Group 2 was the control group and comprised 45 different patients (149 sera) who did not have clinical or microbiological data to document invasive candidiasis (Table 2). Only sera to be studied by IFA were adsorbed previously with heat-killed blastospores of *C. albicans *(see below).

### Rabbit antiserum

A polyclonal antiserum against the purified recombinant N-Hwp1 fragment was obtained in a New Zealand White rabbit immunized subcutaneously with a suspension containing 0.2 mg of protein in 0.5 ml of saline and 0.5 ml of complete Freund's adjuvant. After the first immunization, the rabbit was injected three more times every two weeks with 0.1 mg of protein in 0.5 ml of saline and 0.5 ml of incomplete Freund's adjuvant. A preimmune serum was obtained before immunization, and samples of immune serum were extracted weekly from the ear marginal veins of the rabbit. Pre-immune serum did not react with the recombinant Hwp1 fragments by immunoblotting. The animal study was performed in accord with the guidelines of the Department of Agriculture of the Basque Government.

### Immunoblot assay

Proteins were separated by SDS-PAGE by the method of Laemmli [17] in a minigel system. Electrophoresis of the purified Hwp1 recombinant fragments was done in 10% acrylamide gels at 200 V. The proteins were transferred to Immobilon^® ^membranes (Millipore Ibérica, Madrid, Spain) and incubated with human sera diluted 1:100 in 0.1 M Tris-buffered saline (TBS) supplemented with 8% nonfat dry milk pH 7.2 for 1 h at 37°C with slow agitation. After three washes with TBS, the membranes were incubated with horseradish-peroxidase (HRP) conjugated anti-human immunoglobulin G (IgG) antibodies (Sigma) for 1 h at 37°C. They were washed again and the immunoreactive bands were visualized with 4-chloro-1-naphtol (Sigma). After visual inspection of the blots, sera were qualified as positive if a band of 36–38 kDa was present.

A similar protocol was used with the rabbit anti-N-Hwp1 serum and the mouse monoclonal antibodies anti-HSV (Novagen) and anti-c-Myc (Exbio Praha, Vestec, Czech Republic) used to identify the recombinant proteins. In these cases, the HRP-conjugated secondary antibodies were directed against rabbit IgG (Sigma) and mouse IgG (Sigma), respectively.

### Indirect immunofluorescence assay

It was carried out essentially as previously described [18]. Antibodies to CAGT were detected by an indirect immunofluorescence assay (Candida IFA IgG, Laboratorios Vircell S.A., Granada, Spain). Briefly, every serum was adsorbed with 19 volumes of heat-killed blastospores of *C. albicans *NCPF 3153 in order to remove antibodies against the blastospore cell wall surface and allow the detection of germ tube surface specific antibodies.

Adsorbed sera and its serial double dilutions were applied to each well of the immunofluorescence slides and incubated at 37°C for 30 min in a wet chamber. The slides were washed in PBS and incubated with FITC-conjugated goat anti-human IgG at 37°C for another 30 min in a wet chamber. The slides were washed again and examined with a microscope equipped for epifluorescence. The titer assigned to each serum was the highest dilution of the serum showing fluorescence on the entire *C. albicans *germ tube cell wall surface. Patients were judged positive when reverse titers of CAGT were 160 in at least one serum sample.

### ELISA

Preliminary checkerboard titration experiments were performed to determine optimal concentration of the antigen, by comparing known positive and negative human sera. Microtiter vinyl plates (Costar, Cambridge, Mass, USA) were coated with the purified recombinant N-Hwp1 fragment (0.3 μg/ml). The antigen was diluted in 0.05 M sodium carbonate buffer (pH 9.6) and a 100 μl volume was added to each well and it was incubated at 4°C overnight. After coating, the wells were washed once with PBS and blocked with 100 μl of PBS supplemented with 1% bovine serum albumin (PBSB) at 37°C for 1 h. Sera were diluted 1:1000 in PBSB supplemented with 0.05% Tween 20 (PBSBT) and assayed in triplicate (100 μl/well). After 1 h of incubation at 37°C, the wells were washed three times with PBSBT. A volume of 100 μl of a 1:1000 dilution of HRP-conjugated human anti IgG (Sigma) in PBSBT was added to each well and the plates were incubated at 37°C for 1 h. The plates were then washed three times with PBSBT followed by the addition of 100 μl of the substrate solution, containing 0.05 M citric acid, 0.1 M *di*-sodium hydrogen phosphate anhydrous, 0.05 % of 30 % hydrogen peroxide and 0.05 % *o*-phenylenediamine (Sigma) to each well. The plates were incubated at room temperature for 30 min in darkness. The reaction was stopped by the addition of 50 μl per well of 0.5 M sulphuric acid and the absorbance was measured at 490 nm using an automated ELISA plate reader (Microplate Autoreader, Bio-Tek Instruments, Montpelier, Vermont, USA). Results were expressed as a relative absorbance index calculated by dividing the absorbance of the sample by the absorbance of a reference serum.

### Statistics

Sensitivity, specificity, and positive and negative predictive values were calculated as described by Kozinn et al. [19]. Mean values of relative absorbance of both groups were compared by the t test (Microsoft Excel), values of P < 0.05 were considered statistically significant.

## Authors' contributions

AL, NE and SB carried out the molecular constructions and performed the analysis of the serum samples. JCGR and AdP collected the sera and performed the clinical evaluations of the patients, correlating clinical and serological data. MDM supervised the serological study and helped draft the manuscript. JP designed the study and wrote the manuscript. All authors have read and approved the final manuscript.

## Figures and Tables

**Table 1:** Clinical and serological characteristics of patients with proven invasive candidiasis (group 1).

Patient No.	Underlying condition	*Candida *spp. isolated in blood	Reactivity by immunoblotting	Highest titer of antibodies to CAGT	Reactivity by ELISA
1	Polytraumatized suprarenal insuficiency	*C. albicans*	+	5120	+
2	Bladder surgery	*C. albicans*	+	20480	+
3	Abdominal surgery, Crohn's disease	*C. albicans*	-	1280	+
4	Polytraumatized	*C. albicans*	-	1280	+
5	Heart surgery, mediastinitis	*C. tropicalis*	-	160	+
6	Pneumonia	*C. utilis*	-	1280	+
7	Guillain-Barré syndrome	*C. albicans*	+	20480	+
8	Polytraumatized	*C. albicans*	+	5120	+
9	Abdominal surgery	*C. tropicalis*	-	160	+
10	Abdominal surgery	*C. albicans*	-	1280	-
11	Pancreatitis	*C. glabrata *and *C. albicans*	+	1280	+
12	Abdominal surgery	*C. albicans*	+	5120	+
13	Colon surgery	*C. albicans*	-	640	+
14	HIV	*C. dubliniensis*	-	320	+
15	Abdominal surgery	*C. parapsilosis*	-	640	+
16	Pneumonia	*C. parapsilosis*	-	80	+
17	Pancreatitis	*C. glabrata*	+	640	+
18	Asthma	*C. parapsilosis*	-	320	+
19	Intestinal occlusion	*C. albicans*	-	20	+
20	Cirrhosis, hepatitis B	*C. albicans*	-	1280	+
21	Abdominal surgery, ulcerative colitis	*C. albicans*	+	320	+
22	Surgery, ulcerative colitis	*C. albicans*	-	640	+
23	Surgery, ulcerative colitis	*C. guilliermondii*	-	20	-
24	Rheumatoid arthritis, immunosuppressors	*C. parapsilosis*	-	320	+
25	Renal transplant, immunosuppressors	*C. albicans*	-	20	+
26	Burn	*C. parapsilosis*	-	20	+
27	Burn	*C. albicans*	-	2560	+
28	Burn	*C. albicans*	-	320	+
29	Abdominal surgery	*C. albicans*	-	160	+
30	Acute lymphocytic leukemia	*C. albicans*	-	20480	+
31	Acute myeloid leukemia	*C. albicans*	+	20480	+
32	Non-Hodgkin's lymphoma	*C. parapsilosis*	-	5120	+
33	Acute myeloid leukemia	*C. krusei*	-	0	-
34	Hepatosplenic candidiasis	*C. albicans*	-	1280	+
35	Chronic lymphocytic leukemia	*C. albicans*	-	20	-
36	Acute myeloid leukemia	*C. albicans*	+	320	+

**Table 2:** Clinical and serological characteristics of patients without invasive candidiasis (group 2).

Patient No.	Underlying condition	Microorganism isolated/specimen	Reactivity by immunoblotting	Highest titer of antibodies to CAGT	Reactivity by ELISA
37	Leukemia	*S. prolificans*/Blood	-	0	-
38	Heart surgery	*A. fumigatus*/Necropsy	-	80	+
39	Heart surgery	*A. fumigatus*/Valvular prosthesis	-	0	-
40	Heart surgery	*A. baumannii*/Bronchial brush	-	80	+
41	Multiorgan failure	*E. coli*/Blood	-	20	-
42	Herpetic encephalitis		-	40	-
43	Brain surgery	*A. baumannii*/CSF	-	0	-
44	Abdominal surgery	*E. coli*/Abdominal drain	-	20	-
45	Craneal trauma	*P. aeruginosa*/Bronchial aspirate	-	80	-
46	Cervical trauma	*S. epidermidis*/Venous catheter	-	0	-
47	Pancreatitis		-	0	-
48	Abdominal surgery	*A. baumannii*/Blood	-	20	-
49	Pancreatitis		-	0	-
50	Pancreatitis		-	80	-
51	Heart surgery		-	0	-
52	Acute respiratory insuficiency	*S. pneumoniae*/Blood	-	40	+
53	Polytrauma	*E. coli*/Abdominal drain	-	0	+
54	Craneal trauma	*P. aeruginosa*/Blood	-	0	-
55	Chronic obstructive pulmonary disease	*A. baumannii*/Bronchial brush	-	0	-
56	Heart surgery	*S. epidermidis*/Venous catheter	-	0	+
57	Abdominal surgery	*S. epidermidis*/Venous catheter	-	80	-
58	Craneal trauma	*A. baumannii*/Blood	-	80	-
59	Pancreatitis	*E. faecalis*/Pancreatic drain	-	80	-
60	Acute respiratory insuficiency	*H. influenzae*/Bronchial brush	+	160	-
61	Heart surgery	*S. epidermidis*/Venous catheter	-	0	+
62	Cirrhosis	*P. mirabilis*/Venous catheter	+	0	-
63	Peritonitis	*E. coli*/Abdominal drain	-	0	-
64	Colon surgery	*A. baumannii*/Abdominal wound	-	0	-
65	Polytrauma	*A. baumannii*/Blood	-	80	-
66	Aplastic anemia		-	20	-
67	Acute myeloid leukemia		-	80	+
68	Acute myeloid leukemia		-	0	-
69	Aplastic anemia		-	0	+
70	Acute lymphocytic leukemia		-	0	-
71	Chronic lymphocytic leukemia		-	40	-
72	Allogeneic BMT		-	20	-
73	Acute myeloid leukemia		-	0	-
74	Acute myeloid leukemia		-	0	-
75	Acute myeloid leukemia	Coagulase negative *Staphylococcus*/Blood	-	0	-
76	Myelodysplastic syndrome	*Aspergillus *sp./Necropsy	-	160	-
77	Acute myeloid leukemia (Allogeneic BMT)	*Aspergillus *sp./Necropsy	-	160	-
78	Cronic lymphocytic leukemia	*P. jiroveci*/BAL	-	20	-
79	Acute myeloid leukemia	*Aspergillus *sp./Necropsy	-	1280	-
80	Acute myeloid leukemia	*A. flavus*/BAL	-	40	-
81	Acute myeloid leukemia	*Aspergillus *sp./Necropsy	-	20	-

CSF: cerebrospinal fluid; BMT: bone marrow transplantation; BAL: bronchoalveolar lavage.
